# Partial Antiviral Activities Detection of Chicken *Mx* Jointing with Neuraminidase Gene (NA) against Newcastle Disease Virus

**DOI:** 10.1371/journal.pone.0071688

**Published:** 2013-08-16

**Authors:** Yani Zhang, Dezhi Fu, Hao Chen, Zhentao Zhang, Qingqing Shi, Ahmed Kamel Elsayed, Bichun Li

**Affiliations:** 1 .Institute of Animal Breeding and Genetics, Provincial Key Laboratory of Molecular Design/College of Animal Science and Technology, Yangzhou University, Yangzhou, China; 2 The First Affiliated Hospital of Soochow University, Suzhou, China; 3 College of Veterinary Medicine, Suez Canal University, Ismailia, Egypt; National Institute for Viral Disease Control and Prevention, CDC, China, China

## Abstract

As an attempt to increase the resistance to Newcastle Disease Virus (NDV) and so further reduction of its risk on the poultry industry. This work aimed to build the eukaryotic gene co-expression plasmid of neuraminidase (NA) gene and myxo-virus resistance (Mx) and detect the gene expression in transfected mouse fibroblasts (NIH-3T3) cells, it is most important to investigate the influence of the recombinant plasmid on the chicken embryonic fibroblasts (CEF) cells. cDNA fragment of NA and mutant Mx gene were derived from pcDNA3.0-NA and pcDNA3.0-Mx plasmid via PCR, respectively, then *NA a*nd *Mx* cDNA fragment were inserted into the multiple cloning sites of pVITRO_2_ to generate the eukaryotic co-expression plasmid pVITRO_2_-Mx-NA. The recombinant plasmid was confirmed by restriction endonuclease treatment and sequencing, and it was transfected into the mouse fibroblasts (NIH-3T3) cells. The expression of genes in pVITRO_2_-Mx-NA were measured by RT-PCR and indirect immunofluorescence assay (IFA). The recombinant plasmid was transfected into CEF cells then RT-PCR and the micro-cell inhibition tests were used to test the antiviral activity for NDV. Our results showed that co-expression vector pVITRO_2_-Mx-NA was constructed successfully; the expression of *Mx* and *NA* could be detected in both NIH-3T3 and CEF cells. The recombinant proteins of *Mx* and *NA* protect CEF cells from NDV infection until after 72 h of incubation but the individually mutagenic Mx protein or NA protein protects CEF cells from NDV infection till 48 h post-infection, and co-transfection group decreased significantly NDV infection compared with single-gene transfection group (*P*<0. 05), indicating that Mx-NA jointing contributed to delaying the infection of NDV in single-cell level and the co-transfection of the jointed genes was more powerful than single one due to their synergistic effects.

## Introduction

Avian influenza viruses (AIV) are enveloped, segmented and negative-stranded RNA viruses, which circulate in world-wide and have caused the biggest concern bird flu and severe poultry industry economic losses [1]. The viruses can be divided into different subtypes based on two surface glycoproteins: hemagglutinin (HA) and neuraminidase (NA). NA gene encoding the neuraminidase protein that is a target antigen of humoral immune, which could induce specific antibodies and inhibit virus release from the infected cells, thereby reduce the virus proliferation and increase the immune protection function [2].

Moreover, sialic acid on the host cell membrane is the main receptor of influenza and NDV. These viruses only bind with that receptor causing lesions on the host infected cell. However, NA can degrade the sialic acid receptor and protect the cells from AIV and NDV infection [3]. So, NA has become the active research field due to its role against influenza virus.

It was reported that, form respiratory infection characteristics of influenza virus, the previously immunized mouse with the recombinant virus could produce serum IgG and IgA antibodies. Also, IgA could be locally detected in the mouse respiratory indicating that the recombinant adenovirus induced a good immune response [4]. The survival rates of the mouse immunized by NA alone and NA and HA together were 75% and 100%, respectively [5]. In a BALB/C mice model, HA and matrix protein could protect mice effectively from flu infection; but combination of HA, NA and matrix proteins could provide the best immune protection for the mice [6].

NA had also the antiviral activities against NDV at early stage of viral infection [7]. Most importantly, the recombinant fowl pox virus of HA-NA genes in chickens were able to express foreign genes steadily and induced specific antibodies; co-expression of NA and HA could protect the chicken from the subtypes HPAIV lethal attack of H5N1 and H7N1[8].

The type I interferon (IFN) response is one of the important systems for anti-viral defense. The Mx proteins belong to the superfamily of dynamin-like, large GTPases that associate with intracellular membranes and are involved in a wide range of intracellular transport processes [9], [10]. It has been reported that Mx proteins exhibit similar biophysical features to that of dynamin, including the propensity to self-assemble into ring-like and helical structures, the ability to tubulate lipids [11], and the large GTPases that possess mechano-chemical function [12]. Importantly, the GTP-binding domain at the N-terminal of the Mx protein is essential for the anti-viral activity [13]. The leucine-zipper motif at the C-terminal is the region for protein–protein interaction that determines the anti-viral specificity [13]. Anti-viral mechanism of human Mx proteins was well studied in mammalian system.

Our previous study has demonstrated that the mutation of chicken Mx cDNA from Ser631 to Asn631 can delay and resist NDV infection [14]. However, we are unclear about the mechanisms of combinational function of Mx and NA. Therefore, the purpose of this study is to evaluate the antiviral activities of NA and Mx combination, so as to give a new insight for virus infection resistance.

## Materials and Methods

### Cells, Vectors, Antibodies and Viruses

Primary CEF cells were prepared from 9–10 d embryos of Suqin Yellow chicken (one of Chinese local breed) and cultured using Dulbecco’s Modified Eagle’s Medium (Gibco) supplemented with 10% (v/v) fetal bovine serum, 100 IU/mL ampicillin and 100 µg/mL streptomycin. The culture condition was maintained in 5% of CO_2_ and humidified atmosphere at 37°C. Cells were passaged every 2∼3 days. Eukaryotic expression vector pcDNA3.0 (Invitrogen) was used to express the chicken *Mx* (the Asn631 genotype) and *NA* and *Mx-NA* cDNAs under the control of cytomegalovirus (CMV) promoter and bovine growth hormone (BGH) poly A signal. NA and Mx antibody were provided by Dr. Wenbo Liu at the College of Veterinary Medicine, Yangzhou University; pathogenic NDV F48E8 strain was provided by Dr. Guoqiang Zhu at the College of Veterinary Medicine, Yangzhou University.

### Vector Construction

**Figure 1:**
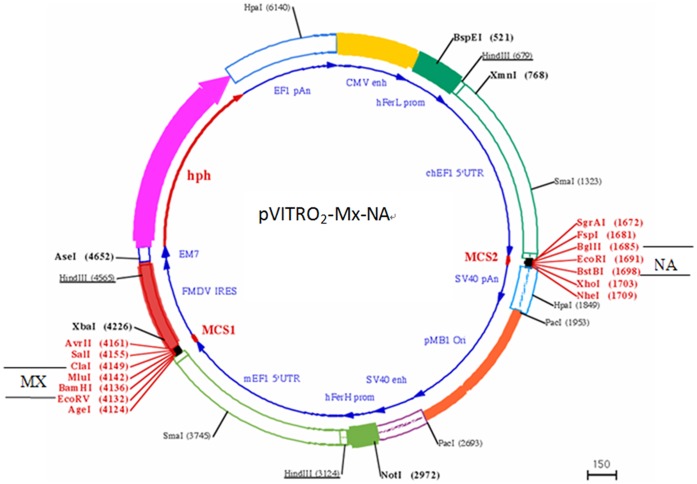
The plasmid profile of pVITRO_2_-Mx-NA. Plasmid pVITRO_2_-hygro-mcs was conducted, then Mx was inserted into MCS1*(Eco*R V and *Cla* I) and NA was insereted into the MCS2(*Eco*R I and *Xho* I restriction site).

*NA* gene (A/Ck/YN/115/2004(H5N1) was amplified by using the primer pair Forward: 5′-CCGCCGGAATTCATGAATCCAAATCAAAAG-3′ and Reverse: 5′-CCGCCGCTCGAGCTACTTGTCAATGGTGAATG-3′ with the *Eco*R I and *Xho* I restriction site. Mx gene was amplified by 5′-GCCCGATATCATGAACAATCCACGGTCCAAC-3′ and 5′-CCGGATCGATCTACAGAGACTTAAAGTC-3′ with the *Eco*R V and *Cla* I restriction site. Then, pVITRO_2_-NA, pVITRO_2_-Mx and pVITRO_2_-Mx-NA were constructed, respectively. The plasmid construction process of pVITRO_2_-Mx-NA was shown in Fig. 1.

### Cell Transfection

CEF and/or NIH 3T3 cells were cultured till 90% confluence in T25 flasks. After one time washing with PBS, cell monolayer was digested with trypsin and the detached cells were suspended in the hypoosmolar buffer system (pH = 7.2) (1 × 10^6^ cells/mL) for electroporation according to the instructions for Multiporator (Invitrogen). Briefly, 375 µL cell suspensions was mixed with 25 µL (1.5 µg) plasmid and transferred into each 2-mm electroporation cuvette. pVITRO_2_ and untransfected cells were used as a negative control. After incubation for 1 min in the electroporation chamber at room temperature, electroporation was performed at 270V for 80 µs. Following incubation for additional 10 min at 4°C, cells were transferred to culture in fresh medium. For virus challenge studies, the transfected cells were subjected to G418 (500 µg/mL, Sigma) selection for two weeks with medium change at every three days.

### Immunofluorescence

At 48 h after transfection, transfected cells were washed one time with PBS and conventional immunofluorescence was performed using the mouse anti-serum (1:800) against chicken Mx protein [8] as the first antibody and FITC-labeled goat anti-mouse IgG (GeneTimes Technology, Inc, Shanghai, China) as the second antibody.

### RT-PCR

G418 selection for two weeks, total RNA was extracted from transfected cells using TRIZOL Reagent (Invitrogen Co., Ltd.) as the manufacturer’s instructions. RT-PCR was performed using PrimeScript® RT reagent Ki (TaKaRa Biotechnology Co., Ltd.), in which reverse transcription was performed in a total volume of 10 µL for 15 min at 37 °C. PCR was performed to amplify NA and Mx genes using the program as follows: initial denaturation (95°C for 8 min), 35 cycles of amplification (95°C for 40 s, 63°C for 45 s and 72°C for 45 s) and the final extension was performed at 72°C for 7 min. 5 µL of RT-PCR products were mixed with 2 µL of loading buffer and subjected to 0.8% horizontal agarose gel electrophoresis. The gels were stained with ethidium bromide for visualization and the amplification results were observed.

### Mini-cytopathic Effect Inhibition Assay

Cytopathic effect inhibition was used to detect the antiviral activities of the Mx-NA protein and the antibodies induced by Mx and NA proteins. This experiment was divided into two groups. For the first group: the CEF cells were transfected with pVITRO_2_-Mx-NA (pVITRO_2_-MN), pVITRO_2_-Mx (pVITRO_2_-M), pVITRO_2_-NA (pVITRO_2_-N) and pVITRO_2_. The transfected cells were subjected to G418 (500 µg/mL, Sigma) selection for two weeks with medium change at every three days. After G418 selection for two weeks, the transfected CEF cells was digested with trypsin and seeded into 6-well plates. After overnight cultivation, 100 TCID50 NDV(F48E8) was added into each well (in triplicates) and incubation was continued for 1 h at 37°C. Cell cultures were observed over 24 h, 48 h, 72 h, 96 h and 120 h of infection for cytopathic effect (CPE) and 50% CPE inhibition was recorded as described. For the second group: the CEF cells were seeded into 6-well plate, the antibodies of Mx-NA(MN group), Mx(M group) and NA(N group) was added into different wells, respectively, after the CEF cells grew into monolayer, and then 100 TCID50 NDV(F48E8) was added into each well (in triplicates). The cells were observed under microscope for cytopathic effect (CPE) and 50% CPE inhibition was recorded as described.

### Virus Titration Assay

G418 selection for two weeks, both the transfected and normal CEF cells, which incubated with the antibody, were digested with trypsin and seeded into 24-well plates. After overnight cultivation, 100 TCID50 NDV(F48E8) was added to each well (in triplicates) and incubation was continued for 1 h at 37°C. After 24, 48 and 72 h infection, the supernatants of the cell cultures were collected for NDV titration on CEF cells. The detail steps were shown as follows: the virus titer of 24 h, 48 h, 72 h, 96 h and 120 h was measured by hemagglutination (HA) titer of each group. HA test was used to detect the erythrocyte agglutination titer of NDV. In this experiment, the micro-method was commonly used and conducted in 96-well plates. Specific experimental operation was as follows: Firstly, 25 μL of sterile saline was added into each well by micropipettes, then 25 μL of NDV antigen solution was put into the first well by micropipettes, the tip was immersed in the liquid slowly and suck up and down for a few times so that the virus diluents mixed with sterile saline completely; then 25 μL of liquid was carefully moved into the second well, so serially diluted to the 11^th^ well, the virus dilution fold was from 1:2 to 1:2048; the 12^th^ well was the erythrocyte as control; The followed step was to add 25 μL of 1.0% erythrocyte suspension. After the mixture was mixed in the shaker for 1–2 min and put it 15 min at room temperature, then the result was observed: The 96-well plates was inclined at 45 °C, the erythrocyte precipitation sinking into the bottom of the tube flowed linearly along the tilt, indicating that the erythrocyte was not or incomplete agglutination; If the erythrocyte paved in the bottom of the well and agglutinated thin layer, the tilt erythrocyte do not flow and demonstrated that the erythrocyte was agglutinated by the virus. The highest dilution fold was to make 100% erythrocyte agglutination, known as the erythrocyte agglutination titer of the virus, the numerical form is 2^n^. In order to facilitate the calculation, the effective value of each group was converted into the integer form by log2x. In this study, single factor analysis of variance method and multiple comparison was used to do analysis of significant difference. The data was analyzed by SPSS 16.0.

## Results

### Expression of Chicken Mx and NA mRNA in NIH 3T3 Cells

To confirm the expression of the chicken Asn631 *Mx* mRNA or *NA* in eukaryotic cells, the expression vector pVITRO_2_-Mx, pVITRO_2_-NA and pVITRO_2_-Mx-NA was transfected into CEF cells. G418 selection for two weeks, total RNA was extracted for RT-PCR using the primer pair to amplify the full-length chicken Mx and NA cDNA. As expected, 2.1 kb and 1.4 kb transcript was amplified in the transfected cells for *Mx* or *NA* respectively, but not showed in the control vector pVITRO_2_-transfected cells. Meanwhile, GAPDH was amplified. All above indicated Mx and NA were expressed in NIH 3T3 cells.

### The Identification of Recombination Mx Protein by Immunofluorescence

**Figure 2:**
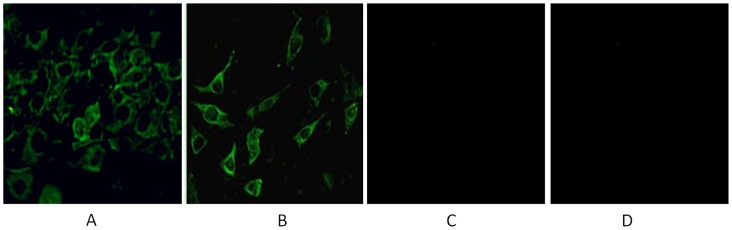
Transient expression of Mx and NA gene in transfected NIH 3T3 cells by IFA. A: The NIH 3T3 cells transfected with pVITRO2-Mx-NA (Mx antibody); B: The NIH 3T3 cells transfected with pVITRO2-Mx-NA (NA antibody). C: The NIH 3T3 cells transfected with pVITRO2; D : NIH 3T3 cells.

To test whether the chicken Asn631 *Mx* and *NA* cDNAs could express correct proteins in eukaryotic cells, the Asn631 *Mx* and *NA* cDNAs were subcloned into the pVITRO_2_ vector and the expression vectors were transfected into NIH 3T3 cells. By using the antiserum against chicken Mx and NA protein, immunofluorescence revealed typical green fluorescence in the cell cultures transfected with pVITRO_2_-Mx-NA (Fig. 2 A and B), but not in the cell culture transfected with the control vector pVITRO_2_ (Fig. 2 C). Meanwhile, it was clear to see that Mx and NA protein mainly distributed at cytoplasm (Fig. 2A and B).

### Antiviral Activities Against NDV of CEF Cells Transfected with pVITRO2-Mx-NA

**Figure 3:**
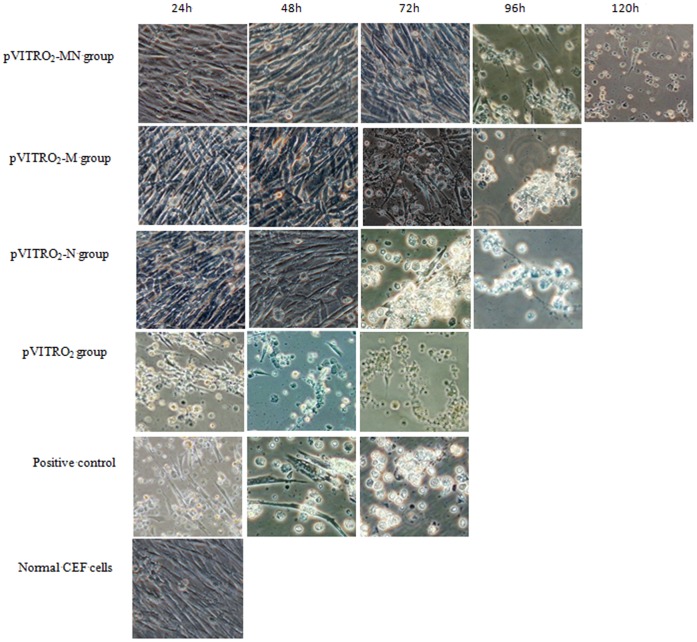
The cell morphology of CEF inoculated NDV for each transfection group at different time (200×). CEF cells were transfected with pVITRO_2_-Mx-NA, pVITRO_2_-Mx, pVITRO_2_-NA and pVITRO_2_. After G418 selection for two weeks, the transfected CEF cells were trypsinized and seeded into 6-well plates. After overnight cultivation, 100 TCID_50_ NDV was added to each well (in triplicates) and incubation was continued for 1 h at 37°C. After 24 h, 48 h, 72 h, 96 h and 120 h infection, cell cultures were observed under microscope for cytopathic effect (CPE) and 50% CPE inhibition was recorded as described. Normal CEF cells infected with NDV was used as a positive control. pVITRO_2_-Mx-NA: pVITRO_2_-MN; pVITRO_2_-Mx: pVITRO_2_-M; pVITRO_2_-NA: pVITRO_2_-N.

To determine the antiviral activities of Mx-NA proteins against NDV, pVITRO_2_-Mx-NA, pVITRO_2_-Mx and pVITRO_2_-NA as well as the control vector pVITRO_2_ were transfected into CEF cells, respectively. G418 selection for two weeks, the transfected cells were challenged with NDV and observed under microscope for CPE over different time points. Similarly, the typical CPE was visible in the pVITRO_2_-transfected cell culture as early as 24 h of post-infection, which was comparable to that in the normal CEF cells culture (Fig. 3). However, the CEF cells transfected with pVITRO_2_-Mx-NA were still close to grow with fibrous morphology until 72 h post-infection, and then the pathological changes emerged (Fig. 3). The CEF cells transfected with pVITRO_2_-Mx or pVITRO_2_-NA were still show the fibrous morphology growing state until 48 h of post-infection, then the cyto-pathological changes appeared (Fig. 3) and the whole cells showed typical pathological changes at 96 h of infection. The cells transfected with pVITRO_2_ or empty plasmid showed pathological changes as early as 12 h after infection, the cells became round, dropped, and the all cells would die after 48 h of infection.

**Figure 4:**
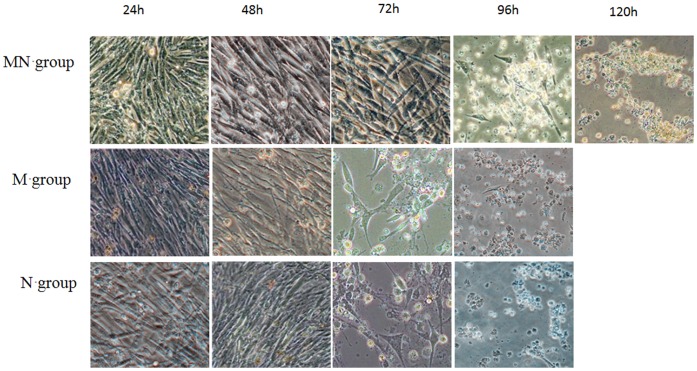
The cell morphology of CEF inoculated NDV for each antibody group at different time (200×). CEF cells were seeded into 6-well plate, the antibody Mx-NA, Mx and NA was add into different well after the CEF cells grew into monolayer, then 100 TCID50 NDV was added into each well (in triplicates). The cell cultures were observed under microscope for cytopathic effect (CPE) at different time. MN group: Mx-NA antibody group; M group: Mx antibody group; N group: NA antibody group.

To confirm the antibodies induced by Mx and NA proteins can also provide cross-protection to NDV, different groups of CEF cells were incubated with Mx-NA, NA and Mx antibody, respectively, and then these cells were challenged by NDV. The results showed the CEF cells was protected by Mx-NA antibody and appeared no pathological changes during 72 h of infection, pathological changes only arose after 72 h of infection and the whole cells showed significant pathological changes after 120 h of infection (Fig. 4). However, there were no pathological changes during 48 h of infection for single Mx or NA antibody, but pathological changes emerged at 72 h of infection and the whole cells showed typical pathological changes after 96 h of infection (Fig.4). These results were consistent with that of the transfection group, which indicate that not only Mx and NA protein could interact with the cells and protect the CEF cells from NDV infection, but also the antibodies induced by Mx and NA proteins could also provide cross-protection to NDV.

**Figure 5:**
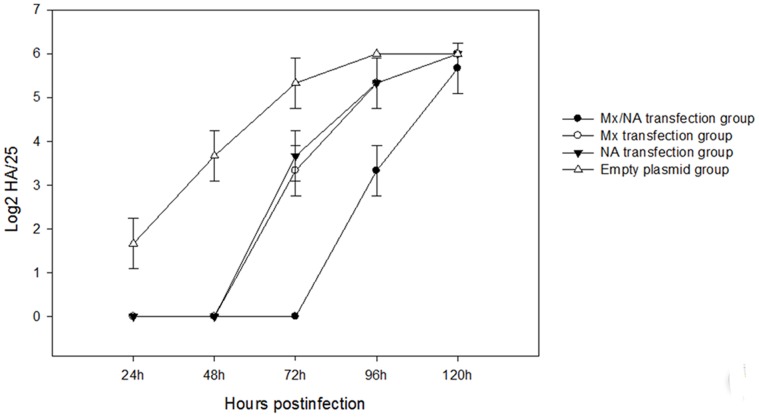
HA of transfection group infected NDV at different time. After G418 selection for two weeks, the transfected CEF cells were trypsinized and seeded into 24-well plates. After overnight cultivation, 100 TCID50 NDV was added to each well (in triplicates) and incubation was continued for 1 h at 37°C. After 24, 48 and 72 h infection, supernatants of the cell cultures were collected for NDV titration on CEF cells.

**Figure 6:**
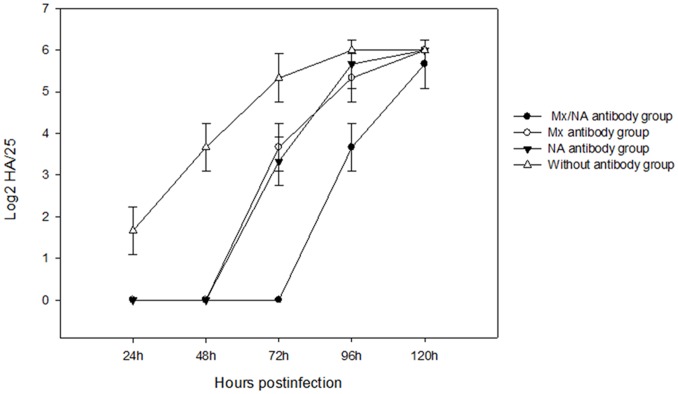
HA of antibody group infected NDV at different time. After G418 selection for two weeks, the antibody incubated CEF cells were trypsinized and seeded into 24-well plates. After overnight cultivation, 100 TCID50 NDV was added to each well (in triplicates) and incubation was continued for 1 h at 37°C. After 24, 48 and 72 h infection, supernatants of the cell cultures were collected for NDV titration on CEF cells.

To compare quantitatively the antiviral activities of the Mx-NA proteins, the supernatants of the above transfected cells and antibody (induced by Mx and NA proteins) group were collected over different time points for NDV titration on CEF cells. As shown in the Fig. 5 and Fig. 6, there was significant difference between the experiment group (the cells transfected with pVITRO_2_-MN, pVITRO_2_-M and pVITRO_2_-N group) and pVITRO_2_ transfected group (*P*<0.05), more importantly, significant difference was found between pVITRO_2_-MN and pVITRO_2_-M or pVITRO_2_-N group, which suggested pVITRO_2_-MN had strong antiviral activities for NDV infection. Also, there were no difference for the three antibody group between 24 h and 48 h (*P*>0.05), but significant difference appeared at 72 h compared to 24 h and 48 h (*P*<0.05).

## Discussion

Although both of NA and Mx gene have the antiviral activities, they have the different antiviral mechanism [14]–[16]. It was reported that molecular biology function of NA protein is to remove the virus particles and sialic acid, which is important for the release of viral particles and prevent the aggregation of the virus particles [17]. NA protein is one of the major surface antigens of the avian influenza virus, which can induce the body to produce specific antibodies as the target antigen of humoral immune and inhibit the virus to release from the infected cells, and then reduce the viral proliferation [18], [19]. It was reported that the incidence of respiratory illness caused by influenza viruses declined with the increase of NA antibody *in vivo*. Since NA antibody inhibits the virus releasing from the cell and infecting other cells, thereby the propagation of the virus is reduced [20]. In a recombinant adenovirus expressing NA of influenza virus, induced immune response was detected [21], in which, the cytopathic effect inhibition assay further showed that CEF cells transfected with NA gene had the anti-NDV activity and CEF cells resisted NDV infection within 48 h, indicating that NA protein was expressed successfully by CEF cells. When CEF cells were infected by the virus, NA protein played the role of the anti-virus by inhibiting the release of the virus and reducing the propagation of the virus. Then we guess: the antiviral activity will be increased in this study if the CEF cells were transfected with pVITRO_2_-Mx-NA or pVITRO_2_-NA followed by the NA antibody incubation.

So far, the antiviral mechanism of Mx protein has been not known completely, although the triple GTP-binding region is an indispensable part for Mx protein and GTP activity is essential for playing its antiviral function [22]. Mx proteins inhibit a variety of negative-strand RNA viruses. Different Mx proteins have different anti-viral specificity and the antiviral mechanisms are also slightly different. Mx protein located in the nucleus can inhibit the replication of the influenza virus in mice. However, Mx A can block the virus into the cytoplasm and prevent viral nucleocapsid getting into the nucleus, and inhibiting virus replication in the nucleus [23]. The anti-VSV mechanism of Mx A protein was to inhibit viral transcription initiation stage by blocking RNA primer and mRNA synthesis. Similarly, cytopathic inhibition assay showed that CEF cells transfected with *Mx* gene demonstrated antiviral activity and CEF cells couldn’t be infected by NDV within 48 h, which indicated that Mx protein was expressed by CEF cells. Mx plays its antiviral activity by inhibition of its replication.

In summary, both Mx and NA proteins have the ability to resist the virus infection, it will be a desirable antiviral pathway if both of genes could be combined together. This study indicated that Mx-NA co-transfection group prolonged significantly the CEF cells resistance against viral infection; there was no pathological change within the first 72 h of infection, which was significantly better than lonely Mx gene group or NA gene group. These results suggest that Mx protein inhibit viral replication while NA proteins prevent the virus releasing from the cell and infecting other cells, thereby reducing the virus propagation and thus enhance anti-virus ability of the cell, demonstrating that there are synergistic antiviral effects between *Mx* gene and *NA* gene. However, the intrinsic antiviral mechanisms between these two genes need to be further explored.
